# Beyond Multilevel Selection in Cancer: Rethinking Metastasis Through Selection for Function

**DOI:** 10.1002/bies.70094

**Published:** 2025-12-03

**Authors:** Frédéric Thomas, Antoine M. Dujon

**Affiliations:** ^1^ CREEC/CANECEV, Mivegec (CREES) Department University of Montpellier, CNRS, IRD Montpellier France; ^2^ School of Life and Environmental Sciences Deakin University Waurn Ponds Victoria Australia

## Abstract

Laplane et al. recently provided a valuable framework for understanding cancer evolution through multilevel selection (MLS), distinguishing between MLS1, where groups differ in persistence based on the traits of their constituent cells but do not reproduce or evolve group‐level adaptations, and MLS2, where groups themselves reproduce and possess emergent fitness distinct from that of individual cells. However, as the authors themselves acknowledge, applying MLS2 to metastasis is challenging for several reasons. We argue that, rather than behaving as isolated evolutionary units, tumor sites function as components of a distributed system. This perspective suggests that metastasis may be better understood through the lens of selection for function, a framework that explains how traits contributing to system‐level persistence can be maintained without requiring group‐level reproduction. This approach complements MLS theory and helps account for the resilience of the metastatic system as a whole, namely, the persistence and coordination of multiple tumor sites functioning as a collective rather than as isolated tumors, beyond classical Darwinian models. It also aligns with the view that metastasis may reflect the reactivation of ancient cellular programs in a novel, nonreproductive context.

## Graphical abstract

Metastasis can be understood not as competing tumor groups but as interconnected parts of a coordinated cancer system. Instead of requiring group‐level reproduction, the “selection for function” framework suggests that certain tumor configurations persist because they enhance system‐wide resilience, communication, and adaptability in the face of pressures such as therapies and immune responses.

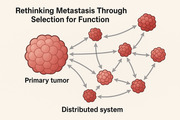

The MLS1/MLS2 framework recently proposed by Laplane et al. [1] provides a valuable structure for examining multilevel cancer dynamics. By distinguishing between selection acting on groups via the traits of their constituent cells (MLS1), which may affect persistence but lacks group‐level reproduction or adaptations, and selection acting on groups as evolutionary units with emergent fitness and reproduction (MLS2), their model offers a clear foundation for exploring how tumor organization may influence evolutionary trajectories. We note that Laplane et al. [1] themselves acknowledge the conceptual and empirical challenges of applying MLS2 to metastasis, explicitly identifying this case as uncertain and difficult to test. Their openness to these limitations reinforces the need to explore complementary frameworks, such as selection for function (see below), that better capture, in our view, the dynamic, nonreproductive, and system‐level nature of metastatic progression.

While the MLS1/MLS2 framework offers a clear and testable foundation for investigating how tumor composition may shape evolutionary trajectories, we contend that its application to metastasis faces substantial empirical and conceptual limitations. Metastatic lesions may exhibit some group‐like features, such as clonal expansion, microenvironmental remodeling, or partial autonomy. However, metastases do not necessarily meet the conditions needed to qualify as MLS2 entities. They lack clear boundaries, do not reproduce as autonomous units, show little developmental consistency, and are not involved in any well‐defined form of competition between groups. In addition, they are often highly variable, can be transient, and remain closely linked to their primary tumor through a web of reciprocal interactions.

The numerous exchanges between tumor sites, through, for instance, the transfer of exosomes, the circulation and reseeding of cancer cells, or the influence on the immune system, suggest that tumors are not isolated collections of separate competing units. Conversely, it seems that they behave more like dynamic, interconnected systems, where different parts evolve in coordination, even if no central control exists. For instance, studies have shown that exosomes from the primary tumor can precondition distant niches for metastasis [2, 3], while circulating metastatic cells can reseed the primary tumor itself, altering its behavior [4, 5, 6]. Such bidirectional feedback contradicts the assumption of autonomous, reproductively isolated tumor groups. Thus, this level of integration makes it relatively difficult to interpret these malignant dynamics solely through classical Darwinian models and points to the need for other theoretical frameworks.

Cancer progression involves the emergence of novel systems arising from extensive genome and karyotype reorganization, producing heterogeneous cellular “species” and continually shifting interaction networks. This supersystem perspective challenges classical microevolutionary interpretations and highlights the importance of integrating genomic architecture into MLS1/MLS2 distinctions. These emergent dynamics align naturally with a selection‐for‐function view (see below), which better captures how metastasis stabilizes system‐level configurations rather than resulting from group‐level reproduction.

The selection for the function framework developed by Wong et al. [7], when adapted to cancer (see [8]), provides such an alternative. Unlike MLS2, it does not require formal reproduction or competition among bounded units. It instead stipulates that certain configurations (e.g., biochemical, structural, or behavioral) can become stabilized within a system because they contribute to its adaptability or persistence. When applied to cancer, this framework would allow, in our view, to interpret metastasis not as the outcome of successful group‐level competition, but as a kind of innovation that enhances the overall tumoral system resilience in the face of ecological and/or therapeutic pressures. The metastatic cascade, for example, may persist not because it guarantees clonal success, but because it contributes, through redundancy, spatial dispersion, and plasticity, to the stability of the overall tumoral system as a whole.

This shift in perspective does not negate the utility of MLS logic, particularly for interpreting clonal selection within tumors (MLS1) or in rare cases where transmissible cancers evolve (as in Tasmanian devils, dogs, bivalves, and hydra, see [9]). However, it suggests that several enigmatic and clinically relevant tumor behaviors, such as metastasis, immune escape, or microenvironmental remodeling, could be better understood through a functionalist lens that emphasizes the stabilization of system‐level configurations (e.g., inter‐tumoral architectures) and their persistence over time. While this view departs from traditional models centered on individual or group‐level competition, it remains compatible with an extended MLS1 framework, in which collectives differing in internal composition or coordination (without reproducing as groups) vary in their capacity to support tumor‐level or system‐level resilience.

From a clinical perspective, this systemic perspective is also consistent with the observation that metastatic cancers are harder to treat not only because of their spatial dissemination, but because of their ability to co‐opt systemic processes, adapt across environments, and maintain resilience through inter‐focal communication. Therefore, we suggest that understanding these behaviors as emergent functions rather than traits selected through classical competition could open new therapeutic avenues aimed at disrupting tumor‐level integration and cooperation (e.g., [10]).

Interestingly, an alternative but non‐mutually exclusive hypothesis worth considering is that the metastatic process may represent the reactivation of evolutionarily ancient cellular programs, originally selected in unicellular or early multicellular contexts (see, for instance [11, 12]). Somatic cells would retain Darwinian reflexes in latent form, and when cancer disrupts the multicellular regulatory framework, these ancient programs could be expressed inappropriately, leading to behaviors, like metastasis, that mimic adaptive strategies but are decoupled from current reproductive logic. From this angle, metastasis might be seen as an atavistic misexpression: a strategy that once made Darwinian sense, but now persists in a different system where its adaptive value must be assessed through a different lens, such as selection for function. This may help explain why metastasis defies straightforward interpretation through classical Darwinian criteria. This perspective opens new avenues for understanding, and ultimately disrupting, the evolutionary dynamics that drive cancer progression.

## Author Contributions

The authors take full responsibility for this article.

## Funding

This work was funded by the CNRS (IRP CANECEV), the HOFFMANN Family, and by the following grant: EVOSEXCAN project (ANR‐23‐CE13‐0007).

## Conflicts of Interest

The authors declare no conflicts of interest.

## Data Availability

The authors have nothing to report.
